# Differences in Preoperative Health-Related Quality of Life between Women Receiving Mastectomy or Breast Conserving Surgery in a Prospectively Recruited Cohort of Breast Cancer Patients

**DOI:** 10.3390/curroncol30010010

**Published:** 2022-12-22

**Authors:** Elaine McKevitt, Maria Saleeb, Guiping Liu, Rebecca Warburton, Jin-Si Pao, Carol Dingee, Amy Bazzarelli, Katelynn Tang, Trafford Crump, Jason M. Sutherland

**Affiliations:** 1 Department of Surgery, Faculty of Medicine, University of British Columbia, 2775 Laurel Street, Vancouver, BC V5Z 1M9, Canada; 2 Providence Breast Centre, Mount Saint Joseph Hospital, 3080 Prince Edward Street, Vancouver, BC V5T 3N4, Canada; 3 Centre for Health Services and Policy Research, University of British Columbia, 2206 East Mall, Vancouver, BC V6T 1Z3, Canada; 4 Department of Surgery, University of Calgary, 2500 University Drive NW, Calgary, AB T2N 1N4, Canada

**Keywords:** breast cancer, health-related quality of life, total mastectomy, breast conserving surgery, partial mastectomy

## Abstract

As rates of total mastectomy rise, the relationships between surgery modality with domains of health-related quality of life is not well understood. This study reports differences in depression, anxiety, pain, and health status among a cohort of women scheduled to receive total mastectomy or breast-conserving surgery. Patient-reported outcomes measured preoperative differences between patients receiving total mastectomy or breast-conserving surgery in a cross-sectional design. Regression analyses was used to model health outcomes and adjust for patient demographics on patient measures. Participants scheduled for total mastectomy were more likely to report more severe symptoms of depression and anxiety. This association was non-significant after adjusting for demographic differences. Younger participants were more likely to be scheduled for total mastectomy. Age was negatively associated with symptoms of depression and anxiety. Screening patients for mental health symptoms may be particularly important among younger patients who were more likely to report depression and anxiety before their surgery and were more likely to receive total mastectomy.

## 1. Introduction

The impact of breast cancer on mental health is substantial, and psychological distress may affect women in all stages of their treatment, with long-lasting effects [1]. Treatment of breast cancer often begins with surgery in the form of either a mastectomy (with or without reconstruction) or a breast-conserving approach including lumpectomy and oncoplastic procedures.

The choice between total mastectomy (TM) and breast conserving surgery (BCS) is based on a variety of patient and provider factors, including size of lesion to size of breast ratio, surgeon recommendation, cancer type, and patient preference [2]. BCS has been shown to have fewer psychological sequelae and improved satisfaction with breasts post operatively compared to TM [3,4] but the introduction of immediate breast reconstruction (IBR) combined with TM has improved psychosocial outcomes for women needing TM [5,6].

Despite strong evidence that both BCS and TM have similar survival outcomes for women, TM is chosen by many women due to the belief that is the safer treatment [2]. The type of breast cancer treatment may be associated with women’s postoperative psychosocial wellness [7]. However, there is limited literature describing symptoms and health differences between the treatment modalities in the preoperative period, nor how to intervene to improve health and surgical outcomes.

Understanding factors leading patients to opt for TM when BCS is feasible creates an opportunity to increase rates of a procedure that has fewer surgical and psychosocial complications. Although some studies have tried to address this complex issue, many focus on shared decision-making processes [8], leaving little information on patient’s physical and mental health at diagnosis. This knowledge is an important first step at exploring why patients may opt for TM, and so that nonoperative services can be tailored to optimize preoperative health and well-being.

Several studies have assessed patients’ health symptoms and satisfaction with breasts post-treatment through patient-reported outcome measures (PROMs); however, most studies assess patients after surgery or focus on a particular surgical approach. There is a paucity of literature evaluating preoperative health-related quality-of-life (HRQoL) more broadly. Understanding women’s preoperative health may help healthcare providers screen and assess those who may be at risk for worse postoperative outcomes, as other studies have down that patients reporting worse symptoms preoperatively are more likely to suffer worse pain, nausea, and fatigue after their breast cancer surgery [9].

To characterize women’s physical and mental health prior to operative treatment, the objective of this study is to describe aspects of women’s health-related quality of life (HRQoL) before surgery for breast cancer and compare differences in medical comorbidities and mental health symptoms between women scheduled for TM or BCS. This study will also explore associations between age, comorbidities and socioeconomic status and ethnocultural differences, factors which have been previously associated with depressive symptoms [10,11], increased pain [12,13], and health status [14,15].

## 2. Materials and Methods

### 2.1. Design

This study is based on a prospectively recruited sample of consecutive patients registered for BCS or TM for the treatment of breast cancer at a single academic teaching hospital site between September 2017 and August 2020. Patients referred for treatment of breast cancer are seen in consultation by one of the six breast cancer surgeons at our center where a decision to proceed with BCS or TM is made, and they are placed on the surgical wait list. Breast reconstruction is discussed with all patients proceeding with TM and interested patients are referred to plastic surgery to discuss and arrange immediate reconstruction.

As an element of ongoing quality improvement initiative in the setting’s health system focusing on perioperative health, all operative patients are invited to complete a survey package. The survey package includes PROMs that measure health status, depression, anxiety, pain and symptoms. BCS and TM patients are also asked to complete the Breast-Q.

A list of patients newly scheduled for BCS or TM for the treatment of breast cancer was provided to the study team. To be eligible, patients had to complete a survey package, be 18 years of age or older, not reside in a conjugate living setting such as a nursing home and be able to communicate in English.

All prospective patients were contacted by phone by the study team. Participants were offered a choice of receiving the survey package through the mail or completing the survey online through a secure website. The survey package included several Patient Reported Outcome (PRO) instruments. Participants’ PROs data was linked with their medical records to accurately identify participants scheduled for mastectomy or breast-conserving surgery.

### 2.2. Patient-Reported Outcomes

#### 2.2.1. Patient-Health Questionnaire-9 (PHQ-9)

The PHQ-9 is a nine-item instrument that measures depression-related symptoms and functional impairment [16]. Each item is scored on a four-point Likert scale and values range from 0 (“Not bothered at all”) to 3 (“Bothered nearly every day.”) The items’ values are summed to determine the instrument’s score. PHQ-9 values of 10 and 15 represent moderate and moderately severe depression, respectively [16].

#### 2.2.2. Generalized Anxiety Disorder (GAD-7)

The GAD-7 is a seven-item instrument that measures respondents’ symptoms of anxiety [17,18]. Each item is scored on a four-point Likert scale, and values range from 0 (“Not at all”) to 3 (“Nearly every day”.) Items are summed to determine the instrument’s score. Values of 10 or higher have been associated with moderate anxiety, while values of 15 or higher have been associated with severe anxiety [17].

#### 2.2.3. Pain Intensity (P), Interference with Enjoyment of Life (E), and Interference with General Activity (G), PEG

The PEG is a three-item instrument that measures pain intensity (one item) and pain interference (two items) [19]. Each item is valued from 0 (no pain/interference) to 10 (as bad as you can imagine). The instrument score is calculated as the average of the three item values.

#### 2.2.4. EuroQoL EQ-5D-5L

The EQ-5D-5L is a measure of health status [20]. The EQ-5D includes a visual analogue scale (VAS), and whose values range between 0 (“the worst health you can imagine”) to 100 (“the best health you can imagine”).

#### 2.2.5. Socioeconomic Status Variables

To measure socioeconomic status (SES), the patient’s address was linked with Statistics Canada’s neighborhood-level measures of deprivation and marginalization, the Canadian Index of Multiple Deprivation [21]. The Canadian Index of Multiple Deprivation measures attributes of residents at each dissemination area level, which is comprised of approximately 140 households [21]. This study used two Canadian Index of Multiple Deprivation indexes: situational vulnerability which measures income-related deprivation, and ethnocultural composition which measures neighborhood ethnic diversity. For each of the two measures, Statistics Canada publishes equally sized quintiles, ranging from ‘least’ to ‘most.’

#### 2.2.6. Breast-Q^TM^

The Breast-Q^TM^ [22,23] is a widely used and validated survey tool developed to evaluate PROMs in breast cancer patients undergoing mastectomy, reconstruction, or BCS. It is scored from 0 (worst) to 100 (best). We used four subscales measuring: expectations of care, psychosocial well-being, sexual well-being, and physical well-being.

### 2.3. Analysis

The demographic characteristics of participants was summarized by counts and percentages, presented for the overall sample and by surgery type. Age was categorized for presentation purposes. A chi-square test was used to test for statistically significant differences in the distribution of participants’ age categories between mastectomy or breast-conserving surgery.

Summary statistics of the PHQ-9 (depression), GAD-7 (anxiety), PEG (pain), EQ-5D VAS (overall health) and Breast-Q^TM^ were summarized for the sample of participants and by surgery type. An analysis of variance was used to measure whether there was a statistically significant difference between mean values of PROs between mastectomy and breast-conserving surgery. As this was an exploratory study of preoperative health and treatment modality, no formal hypotheses were tested.

The count and proportion of participants whose PHQ-9 and GAD-7 values met or exceeded the scales’ treatment thresholds of 10 or 15 were reported for the overall sample and then stratified for mastectomy or breast-conserving surgery. Chi-square tests were used to test for statistically significant differences in the distribution of participants meeting or exceeding the treatment thresholds of 10 or 15 of the PHQ-9 and GAD-7 by surgery type.

Linear regression models were used to measure associations between participant’s PROs and mastectomy or breast-conserving surgery. Each PRO (PHQ-9, GAD-7, PEG, and EQ 5D VAS) were modelled separately. Each model adjusted for participants’ age, and SES variables. A dichotomous variable was included in the models for mastectomy or breast-conserving surgery. All terms were included in the model, irrespective of their statistical significance. Linear assumptions were checked using residual and Q-Q plots.

A subgroup analysis was conducted on participants scheduled for TM. Participants scheduled for mastectomy were stratified into those scheduled for immediate breast reconstruction and those not having immediate breast reconstruction. Differences in PRO values were tested between the two subgroups using a one-way analysis of variance.

All *p*-values < 0.05 were considered significant, and all tests were two-sided. All analyses were conducted with SAS. This study was approved by the University of British Columbia’s Behavioral Research Ethics Board.

## 3. Results

The participation rate among eligible patients was 34%; there were 2091 eligible patients and 671 completed their PROMs. Non-participants were on average 2 years younger than participants (*p* < 0.01; not shown,) though no differences in comorbidities or other factors were identified between participants and non-participants.

In the study group, 443 participants were scheduled to receive BCS and 228 participants were scheduled for TM. Of the planned total mastectomies, 122 were scheduled for immediate reconstruction. As shown in Table 1, most participants were between the age of 60 and 69 years of age. BCS was more common among older participants (*p*-value < 0.01; chi-square test.) Among participants scheduled for TM, the most common age category was 40 to 49 years of age. Participants who report more medical and psychiatric comorbidities were much more likely to have TM, 39% (90/228) versus 19% (85/443) (*p* < 0.01).

Table 2 shows the summary statistics of the participants’ PROMs. Participants scheduled for TM tended to have higher PHQ-9 and GAD-7 scores than those participants scheduled for BCS (*p*-value = 0.03 and 0.01, respectively.) There were no statistically significant differences in mean PEG (pain) or EQ-5D VAS (overall health) values between TM or BCS.

As shown in Table 3, over 13% (125/671) of participants reported at least moderate symptoms of depression. More participants scheduled for TM reported severe symptoms of depression (*p*-value = 0.04). Over 8% (75/671) of participants reported at least moderate symptoms of anxiety. Severe anxiety was more common among participants scheduled for TM (*p*-value = 0.04).

**Figure 1:**
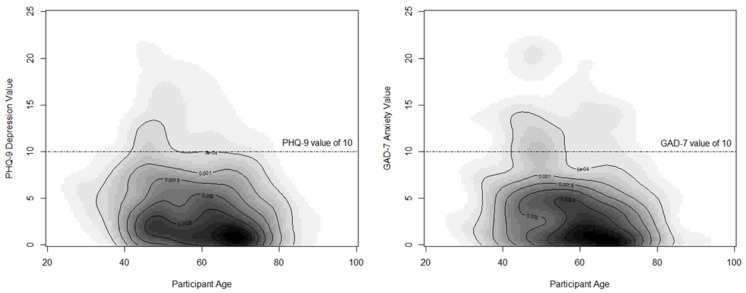
Panel plot of participants reporting symptoms of depression (PHQ-9)^7^ and anxiety (GAD-7) that exceeded clinical thresholds. ^7^ Abbreviation: PHQ-9, Patient-health questionnaire-9; GAD-7, Generalized Anxiety Disorder.

Figure 1 illustrates the relationship between participant’s age and PHQ-9 (depression) and GAD-7 (anxiety) values. For both the PHQ-9 and GAD-7, there was a statistically significant negative relationship between the instruments’ values and participant’s age (*p* < 0.01), as younger participants tended to have higher values on both PROs (worse health).

The regression analyses of Table 4 show that, after adjusting for age, comorbidities and SES differences between groups, there were no differences in either PHQ-9 (depression) or GAD-7 (anxiety) values between the TM or BCS groups. Age was negatively associated with both PHQ-9 (depression) or GAD-7 (anxiety); older participants reported fewer symptoms of depression or anxiety. There were no differences in PEG pain or EQ-5D VAS health status variables between the TM or BCS groups (Table A1 in Appendix A).

Table 5 outlines the results of the Breast-Q^TM^ measure. Similar to results from the PHQ-9, GAD-7 and EQ5D BCS patients report better psychosocial and physical well-being than TM patients and also report better preoperative satisfaction with their breasts.

The subgroup analysis of participants scheduled for immediate breast reconstruction and those not having immediate breast reconstruction found that participants having immediate reconstruction were younger (*p* < 0.01) and had fewer comorbidities (*p* = 0.01) than those not having immediate breast reconstruction. As shown in Table 6, participants having immediate breast reconstruction had higher scores (more symptoms) on the PHQ-9 and GAD-7 and lower (worse health status) on the EQ-5D.

## 4. Discussion

Our study found that women scheduled to receive breast-conserving surgery reported less symptoms of anxiety and depression than patients scheduled for mastectomy, consistent with other findings [3,24,25,26]. However, once adjusting for demographic and socioeconomic characteristics of the study’s members, depression and anxiety were found to be associated with participant’s age, with younger participants more likely to report more symptoms of depression and anxiety and more likely to have total mastectomy. As this is the first study to closely study preoperative HRQoL for breast cancer patients in this way, further study is needed to confirm and explore these findings.

This study was designed as a prospective cohort of consecutive patients scheduled for breast cancer surgery. The goal of this initial study was to explore preoperative HRQoL since there is a paucity of current literature in this population. Although the use of PROs has been increasing since the mid 2000s, different measures are used which makes it difficult to compare results. Efforts are currently underway to describe an ideal set of measures for use in breast cancer treatment [27]. Our institution has been evaluating HRQoL for patients scheduled for nononcologic surgery in multiple surgical specialties. We chose to collect the same general HRQoL measures for this study as had been collected for other surgery types to allow us to make comparisons in future studies to patients undergoing nononcologic surgery to better understand preoperative HRQoL for our breast cancer patients. The data were derived from patient-reported health information and did not include the medical data on indications for procedures. This design was selected to emphasize mental health factors; relationships between HRQoL and tumor factors will be explored in future studies.

Although our study found no difference between planned surgery type and preoperative HRQoL after adjusting for age, studies focusing on postoperative HRQoL have shown that women report better health after BCS. Jay et al. [7] found that BCS was associated with better satisfaction with breasts, sexual well-being and psychosocial well-being. Other postoperative studies support these results, as multiple studies report better HRQoL post-surgery in most or some domains for women receiving BCS [3,7,24,25].

Similar to our findings, studies that have looked at preoperative patients have shown that women scheduled to receive breast cancer treatment report poor sleep quality, symptoms of depression, anxiety, fatigue and pain [9,28,29]. Many women have described the newly diagnosed time period the most difficult and struggled with feeling of despair, hopelessness, and distress [30,31], however these studies focus on time of diagnosis, rather than the preoperative period.

Builes Ramirez [32] used the Breast-Q to evaluate preoperative PROMs in Spain in a group of 112 prospectively recruited patients and found no statistically significant differences between low-score and high-score groups regarding epidemiological and clinical characteristics. This is in contrast to our findings of an association of depression and anxiety with younger age. Our study had a larger sample size but further studies will be needed to further assess relationships between surgical procedure and preoperative HRQoL.

Studies investigating contralateral prophylactic mastectomy have compared preoperative and postoperative PROMs. Lim et al., using the Breast-Q, found that when comparing patients receiving BCS to unilateral and bilateral mastectomy, there was no difference in preoperative breast satisfaction, psychosocial well-being, but a difference in both physical and sexual well-being [33]. Parker found that patients having contralateral prophylactic mastectomy for unilateral breast cancer had increased preoperative worry, distress, and body image concerns compared to patients not having a contralateral prophylactic mastectomy. However, only 26% of patients had BCS which is a lower rate than expected [34].

Many studies have shown the psychosocial benefits to having immediate breast reconstruction with mastectomy [5,33] however, this study’s finding of increased depression and anxiety in patients having immediate reconstruction compared to mastectomy alone is in contrast to this. Patients having reconstruction were younger than those not having reconstruction, which may explain the worse mental health symptoms in this group as our regression analysis suggests preoperative depression and anxiety may be more closely related to age than procedure type. This study site offered breast reconstruction to almost all of our breast cancer patients, which may explain these findings as most patients will have a choice as to whether or not to have reconstruction. This finding may also illuminate which patients may need additional support before their breast cancer surgery, especially since breast cancer patients reporting poor health symptoms before surgery may be at risk for reporting worse postoperative health outcomes [35]. Some studies have shown that increasing education and knowledge in distressed breast cancer patients before their surgery can significantly decrease their anxiety levels before their surgery [36]. 

This study’s observation between younger age and worse mental health could be observed for a variety of reasons. Due to sociocultural norms, breasts are regarded as a symbol of femininity [37]. For many women, losing their breasts may feel as a loss of their womanhood, and decrease their self-esteem [38,39]. These self-negative perceptions may increase a woman’s likelihood of reporting anxiety and depressive symptoms before and after their total mastectomy [39] or express concerns regarding their treatment preferences. These concerns may be more common in younger women.

Understanding the decision to pursue TM when BCS is feasible is complicated, yet important. Why women choose TM when they are safe candidates for BCS is unclear. This is a particularly relevant issue to patients, providers and researchers, as TM rates have been rising [40,41,42,43], despite many studies finding similar survival outcomes between BCS and TM [44,45,46], and BCS being regarded as a better option in many cases [2]. Younger participants in our sample may have been more worried, possibly attributable to larger, more aggressive or recurrent cancer observed among some of younger age [47]. Additionally, it has been recognized that young women with breast cancer have unique care needs. Results from the prospective Canadian study of Young Women with Breast Cancer [48] will further enhance our understanding. The results of this current study evaluating women of all ages, help to provide context for that work.

It is important to acknowledge the limitations of this study. Participation bias is possible, as our analysis indicated that patients that did not participate were 2 years younger on average than participants. Although our response rate was 34%, this rate falls within the range of expected participation of studies of this design. Additionally, this was a cohort study of operative patients, so there may have been heterogeneity in the sample since participation was not limited to stages of disease or treatment. The goal of this study was to understand baseline HRQoL; tumor-related factors and impact on preoperative health, as well as understanding changes in health after surgery, will be analyzed in future studies. Additionally, the majority of data was collected before the COVID-19 pandemic, and pandemic-related symptoms of depression and anxiety were unlikely to have impacted the study. Generalizability to patients in other geographic areas may be difficult, as our patient population was comprised of residents of one province in Canada.

The strengths of this study include a robust sample size, and a population of participants that are demographically diverse. Additionally, our study is the first to look at the association between surgical procedure and preoperative health in this way, leaving room for additional research in this area to identify causal pathways. These findings have suggested that younger patients have higher rates of depression and anxiety and we are working on refining our care pathways to screen for symptoms and offer referral to mental health supports if such symptoms are identified.

## 5. Conclusions

Younger breast cancer participants were more likely to report symptoms of depression and anxiety before breast cancer surgery. Screening and preoperative referral to mental health providers may offer an opportunity to enhance perioperative care.

## Figures and Tables

**Table 1:** Summary of participant’s age category, overall and stratified by partial mastectomy and total mastectomy.

Patient Characteristic	Overall Sample (N = 671)	Breast-Conserving Surgery (N = 443)	Total Mastectomy (N = 228)	*p*-Value
Age				
<49	27.57%	20.54%	41.34%	<0.01
50–59	23.25%	20.99%	27.63%	
60–69	27.87%	34.31%	15.35%	
>70	21.31%	24.15%	15.79%	
Charlson Index				
0	16.55%	18.57%	12.21%	<0.01
1–2	57.66%	62.14%	48.09%	
3+	25.79%	19.29%	39.69%	
SES ^1^—Situational vulnerability				
Q1 Least Vulnerable	27.75%	28.54%	26.22%	0.86
Q2	21.57%	22.15%	20.44%	
Q3	19.61%	19.41%	20.00%	
Q4	20.51%	20.09%	21.33%	
Q5 Most Vulnerable	10.56%	9.82%	12.00%	
SES—Ethno-cultural composition				
Q1 Least Diverse	3.32%	2.97%	4.00%	<0.01
Q2	5.73%	5.94%	5.33%	
Q3	15.84%	19.41%	8.89%	
Q4	38.76%	38.36%	39.56%	
Q5 Most Diverse	36.35%	33.33%	42.22%	

^1^ Abbreviation: SES, socioeconomic status.

**Table 2:** Patient-reported outcome statistics presented for the overall sample and stratified by breast-conserving surgery and mastectomy.

Patient-Reported Outcome	Overall		Breast-Conserving Surgery		Total Mastectomy		F-Stat
	Mean	SD	Mean	SD	Mean	SD	*p*-Value
PHQ-9 ^2^	4.64	4.7	4.36	4.48	5.18	5.06	0.03
GAD-7	4.66	4.96	4.17	4.5	5.73	5.72	<0.01
PEG	1.64	2.25	1.58	2.21	1.77	2.31	0.31
EQ-5D VAS	73.96	18.29	74.82	17.96	72.26	18.84	0.08

^2^ Abbreviation: PHQ-9, Patient-health questionnaire-9; GAD-7, Generalized Anxiety Disorder; PEG, Pain intensity, interference with enjoyment of life, and interference with general activity; EQ-5D VAS, EuroQoL 5-dimension visual analogue scale.

**Table 3:** Number and proportion of participants reporting symptoms of depression (PHQ-9) and anxiety (GAD-7) that exceeded clinical thresholds. Sample size equals 671 participants.

Patient-Reported Outcome	Overall		Breast-Conserving Surgery		Total Mastectomy		Chi-Sq
	N	%	N	%	N	%	*p*-Value
PHQ-9 ^3^ (depression)							
≥10, Moderate	89	13.26%	55	12.42%	34	14.91%	0.36
≥15, Severe	36	5.37%	18	4.06%	18	7.89%	0.03
GAD-7 (anxiety)							
≥10, Moderate	56	8.35%	33	7.45%	23	10.09%	0.24
≥15, Severe	19	2.83%	8	1.81%	11	4.82%	0.02

^3^ Abbreviation: PHQ-9, Patient-health questionnaire-9; GAD-7, Generalized Anxiety Disorder.

**Table 4:** Results of regression analyses of PHQ-9 values and GAD-7 values, adjusting for participant’s age, comorbidities, procedure, and SES.

Regression Effect	PHQ-9 (Depression)			GAD-7 (Anxiety)		
	Estimate	Standard Error	*p*-Value	Estimate	Standard Error	*p*-Value
Intercept	9.87	1.00	<0.01	9.48	1.25	<0.01
Age (Years)	−0.08	0.01	<0.01	−0.09	0.01	<0.01
Charlson Index						
0	−0.32	0.84	0.69	−0.35	1.06	0.73
1–2	−0.62	0.64	0.33	−0.32	0.74	0.66
3+	Reference					
Surgery type						
Breast-conserving surgery	−0.17	0.40	0.67	−0.74	0.50	0.14
Total mastectomy	Reference			Reference		
SES ^4^—Situational vulnerability						
Q1 Least Vulnerable	−0.33	0.71	0.63	0.64	0.93	0.49
Q2	0.26	0.69	0.70	0.77	0.89	0.38
Q3	0.80	0.68	0.23	1.15	0.86	0.18
Q4	0.02	0.67	0.96	0.80	0.85	0.34
Q5 Most Vulnerable	Reference			Reference		
SES—Ethno-cultural composition						
Q1 Least Diverse	−0.02	1.02	0.98	0.42	1.32	0.74
Q2	0.57	0.82	0.48	1.38	1.02	0.17
Q3	0.30	0.61	0.61	−0.43	0.78	0.58
Q4	0.44	0.46	0.32	0.91	0.58	0.11
Q5 Most Diverse	Reference			Reference		

^4^ Abbreviation: PHQ-9, Patient-health questionnaire-9; GAD-7, Generalized Anxiety Disorder.

**Table 5:** Patient-reported outcome statistics presented for the Breast-Q^TM^ stratified by breast-conserving surgery and mastectomy.

Breast-Q^TM^ Scale:	Breast-Conserving Surgery		Total Mastectomy		
	Mean	SD	Mean	SD	*p*-Value
Satisfaction with breasts	67	21.9	62.5	22.2	0.014
Psychosocial well-being	72.8	19.9	68.4	21.9	0.011
Physical well-being	79.2	15.8	74.2	15.9	<0.001
Sexual well-being	57.1	24.1	54.2	25.9	0.21

**Table 6:** Patient-reported outcome statistics presented for the total mastectomy subgroup stratified by receipt of immediate breast reconstruction.

Patient-Reported Outcome	Total Mastectomy with Immediate Breast Reconstruction		Total Mastectomy Alone		
	Mean	SD	Mean	SD	*p*-Value
PHQ-9 ^2^	6.03	5.55	3.84	3.85	<0.01
GAD-7	6.45	6.02	4.42	4.94	0.07
PEG	1.63	2.22	1.98	2.45	0.25
EQ-5D VAS	74.44	17.61	68.92	20.24	0.03

^2^ Abbreviation: PHQ-9, Patient-health questionnaire-9; GAD-7, Generalized Anxiety Disorder; PEG, Pain intensity, interference with enjoyment of life, and interference with general activity; EQ-5D VAS, EuroQoL 5-dimension visual analogue scale.

## Data Availability

All data generated or analysed during this study are available in anonymized format from Jason M. Sutherland.

## Appendix A

**Table A1:** Results of regression analyses of PEG pain values and EQ-5D VAS health status values, adjusting for participant’s age, comorbidities, procedure and SES.

Regression Effect	PEG (Pain)			EQ-5D VAS (Health Status)		
	Estimate	Standard Error	*p*-Value	Estimate	Standard Error	*p*-Value
Intercept	2.57	0.49	<0.01	67.3	3.97	<0.01
Age (Years)	−0.01	0.01	0.68	0.05	0.05	0.32
Charlson Index						
0	−0.21	0.39	0.58	6.65	2.87	0.02
1–2	−0.18	0.27	0.51	4.33	2.25	0.05
3+	Reference			Reference		
Surgery type						
Breast Conserving Surgery	−0.12	0.19	0.52	1.38	1.59	0.38
Total mastectomy	Reference			Reference		
SES ^5^—Situational vulnerability						
Q1 Least Vulnerable	−0.97	0.35	<0.01	0.88	2.88	0.75
Q2	−0.5	0.34	0.14	−0.43	2.83	0.87
Q3	−0.71	0.33	0.03	−1.36	2.77	0.62
Q4	−0.94	0.33	<0.01	−1.08	2.72	0.69
Q5 Most Vulnerable	Reference			Reference		
SES—Ethno-cultural composition						
Q1 Least Diverse	−0.04	0.5	0.92	0.01	4.11	0.99
Q2	0.56	0.4	0.16	−4.52	3.3	0.17
Q3	0.12	0.3	0.67	−2.2	2.46	0.37
Q4	0.32	0.22	0.15	−0.69	1.86	0.7
Q5 Most Diverse	Reference			Reference		

^5^ Abbreviation: SES, socioeconomic status; PEG, Pain intensity, interference with enjoyment of life, and interference with general activity; EQ-5D VAS, EuroQoL 5-dimension visual analogue scale.
